# Trends in Remote Health Care Consumption in Sweden: Comparison Before and During the First Wave of the COVID-19 Pandemic

**DOI:** 10.2196/33034

**Published:** 2022-02-02

**Authors:** Veronica Milos Nymberg, Lina Maria Ellegård, Gustav Kjellsson, Moa Wolff, Beata Borgström Bolmsjö, Thorne Wallman, Susanna Calling

**Affiliations:** 1 Center for Primary Health Care Research Department of Clinical Sciences Malmö Lund University Malmö Sweden; 2 Department of Economics Lund University Lund Sweden; 3 Department of Business Administration Kristianstad University Kristianstad Sweden; 4 Department of Economics University of Gothenburg Gothenburg Sweden; 5 Family Medicine & Preventive Medicine Section Public Health & Caring Sciences Uppsala University Uppsala Sweden; 6 Center for Clinical Research Sörmland Uppsala university Eskilstuna Sweden

**Keywords:** remote health care, telemedicine, primary health care, respiratory tract infections, COVID-19

## Abstract

**Background:**

Remote assessment of respiratory tract infections (RTIs) has been a controversial topic during the fast development of private telemedicine providers in Swedish primary health care. The possibility to unburden the traditional care has been put against a questionable quality of care as well as risks of increased utilization and costs. The COVID-19 pandemic has contributed to a changed management of patient care to decrease viral spread, with an expected shift in contact types from in-person to remote ones.

**Objective:**

The main aim of this study was to compare health care consumption and type of contacts (in-person or remote) for RTIs before and during the COVID-19 pandemic. The second aim was to study whether the number of follow-up contacts after an index contact for RTIs changed during the study period, and whether the number of follow-up contacts differed if the index contact was in-person or remote. A third aim was to study whether the pattern of follow-up contacts differed depending on whether the index contact was with a traditional or a private telemedicine provider.

**Methods:**

The study design was an observational retrospective analysis with a description of all index contacts and follow-up contacts with physicians in primary care and emergency rooms in a Swedish region (Skåne) for RTIs including patients of all ages and comparison for the same periods in 2018, 2019, and 2020.

**Results:**

Compared with 2018 and 2019, there were fewer index contacts for RTIs per 1000 inhabitants in 2020. By contrast, the number of follow-up contacts, both per 1000 inhabitants and per index contact, was higher in 2020. The composition of both index and follow-up contacts changed as the share of remote contacts, in particular for traditional care providers, increased.

**Conclusions:**

During the COVID-19 pandemic in 2020, fewer index contacts for RTIs but more follow-up contacts were conducted, compared with 2018-2019. The share of both index and follow-up contacts that were conducted remotely increased. Further studies are needed to study the reasons behind the increase in remote contacts, and if it will last after the pandemic, and more clinical guidelines for remote assessments of RTI are warranted.

## Introduction

Respiratory tract infections (RTIs) are one of the most common reasons for contacts in Swedish primary care [1]. The outbreak of the COVID-19 pandemic has led to a higher threshold to assess uncomplicated RTIs in primary care using in-person contacts. Thus, the pandemic has catalyzed the development, implementation, and use of remote contacts with primary care providers, including traditional telephone contacts as well as digital contacts (email, chat, video consultations) [2]. Private telemedicine providers offering exclusively video consultations or chat on-demand have been established both in Sweden and worldwide during past years [3,4], and the number of contacts with private telemedicine providers is increasing rapidly [4]. The traditional primary care sector in Sweden has also implemented different telemedicine platforms as a complement to traditional care, including mostly chat or email. Although patients and health care staff report satisfaction with remote contacts [5,6], there is a lack of evidence about risks, benefits, and cost efficiency regarding assessment of symptoms through digital contacts compared with traditional physical contacts in primary care [7-9]. For example, diagnostic difficulties and an increased number of follow-up contacts have been described after management of RTIs by digital contacts in the United States [10]. However, a recent Swedish study found no increase in follow-up rates or antibiotic use for virtual care compared with emergency care or primary care for low-acuity urgent conditions [11]. Previous studies have described the expansion of telemedicine in Sweden before 2019 [12]. In line with international evidence [8], the availability of such services has been associated with a net increase in health care utilization [13].

The COVID-19 pandemic has led to a fast shift in the modality by which patients and health care communicate, from in-person to remote contacts (primarily to minimize the SARS-CoV-2 spread), despite insufficient guidelines and conflicting evidence regarding its impact on health-related outcomes [14]. A basic search in PubMed (MEDLINE) using keywords of the study aim identified a knowledge gap, with no prior studies about how this shift in communication has affected the search pattern for RTIs, and how the composition of follow-up contacts differs if the index contact is physical or digital.

The first aim of this study was to analyze the change in number and percentage of in-person contacts and remote contacts, respectively, for RTI between January-June 2018/19 and January-June 2020. The second aim was to study whether the number of follow-up contacts after an index contact for RTI changed during the study period, and whether the type and number of follow-up contacts differed between index in-person contacts and index remote contacts. A third aim was to study whether the pattern of follow-up contacts differed depending on whether the index contact was with a traditional care provider or a private telemedicine provider.

## Methods

### Study Design

The study design was an observational retrospective analysis describing physician contacts in primary care and at hospital emergency rooms of patients with an index contact for RTIs in January-June 2020, 2019, and 2018. The first 6 months of 2020 were chosen to study the development of RTI-related contacts during the start of the COVID-19 pandemic. The monthly development of physician contacts in 2018 and 2019 was used to illustrate seasonal patterns in the absence of a pandemic. The study was set in the Swedish region Skåne, which is the third largest region (1.4 million inhabitants), with a wide geographical variation including large, middle-sized, and small cities as well as rural areas. The region was relatively mildly hit by the first wave of the pandemic. In the first half of 2020, the number of laboratory-confirmed COVID-19 cases per 100,000 inhabitants was 219; 110 patients with COVID-19 received intensive care and 248 inhabitants died with a COVID-19 diagnosis. This may be compared with one of the worst hit regions in Sweden—Stockholm (2.4 million inhabitants), which recorded 798 confirmed cases per 100,000 inhabitants, 894 intensive care patients, and 2,331 deaths in the same period [15].

### Study Population and Data

The study population consisted of all individuals with a registered address in Skåne (Region Skåne) on December 31 in 2017, 2018, or 2019 (according to the Swedish population register held by Statistics Sweden). For this population, data on in-person or remote contacts with care providers located in Skåne (public or contracting with the region) were collected from the regional health authority’s care register “Region Skånes Vårddatabas” (RSVD). The data included information on date of contact, type of contact (in-person/remote), and up to 8 diagnoses for all care contacts in the period August 2017 to July 2020. Data for the same population located in Skåne on contacts with private telemedicine providers, which are formally located in other regions (Region Sörmland and Region Jönköping) and therefore report to care registers in these regions, were sourced from the health authorities in those regions and a register of extra-regional care contacts of inhabitants in Skåne. Multimedia Appendix 1 presents more details on data sources for the different types of contacts and information about missing data on registered diagnoses from the various sources.

In the analysis, we distinguished between physician contacts that were in-person or remote (ie, consultations by telephone, video, or asynchronous chats). We further distinguished between remote contacts with traditional providers (defined as all primary health care centers and hospital emergency rooms) and remote contacts with pure on-demand telemedicine providers. Such telemedicine services were offered by the private companies Kry, Capio Go, Min Doktor, Doktor.se, Doktor 24, Medicoo, Accumbo. During the study period, traditional care providers mainly offered remote contacts via phone and to some extent by asynchronous chats, whereas private on-demand telemedicine providers primarily offered asynchronous chats or video calls. We linked the data from different registers using pseudo-anonymous individual identifiers provided by Statistics Sweden. The linked data set included all contacts with traditional and private telemedicine providers made by the study population.

### Outcome Variables

Primary outcome variables were *index contacts* and *follow-up contacts with a physician* for patients with a registered RTI-relevant diagnosis (Table 1). Definition of the variables are presented in Table 2.

The study included index contacts occurring during any of the following periods: January 1, 2018-June 30, 2018; January 1, 2019-June 30, 2019; and January 1, 2020-June 30, 2020. We studied the total number of index contacts per 1000 inhabitants, and the total number of follow-up contacts per 1000 inhabitants and per index contact. In addition, we reported the number of unique patients hospitalized with an RTI diagnosis according to Table 1 (per 1000 inhabitants).

**Table 1:** ICD-10^a^ diagnosis codes of relevant diagnoses [16].

Diagnosis code ICD-10	Diagnosis group
J00-J06	Acute upper respiratory infections
J10-J18	Influenza and pneumonia
J20	Acute bronchitis
J22	Unspecified acute lower respiratory infection
R05	Cough
R06.0	Dyspnea
R50	Fever of other and unknown origin
B34.2	Coronavirus infection, unspecified site
B39	Viral infection of unspecified site
B99	Other infectious disease
H65-H70	Otitis media and mastoiditis
U07.1	COVID-19, virus identified
U07.2	COVID-19, virus not identified
ZV100	Health care intervention related to coronavirus infection (ICD-10-SE^b^)

^a^ICD-10: International Classification of Diseases.

^b^ICD-10-SE: the Swedish version of ICD 10.

**Table 2:** Definitions of the outcome variables.

Type of contact	Definition	Subtype of contact
Index contact	The first physician contact with a registered respiratory tract infection–relevant diagnosis after a period of no such diagnosis for at least 181 days.	In-person contacts^a^
		Remote contacts with a traditional provider^b^
		Remote contacts with a private telemedicine provider^c^
A follow-up contact	A physician contact (regardless of diagnosis) within 30 days after the index contact.	In-person contacts^a^
		Remote contacts with a traditional provider^b^
		Remote contacts with a private telemedicine provider^c^

^a^In-person contacts at a primary care center or a hospital emergency room.

^b^Remote contacts with a traditional provider (a primary health care center or a hospital emergency room).

^c^Remote contacts with a private telemedicine provider (offering on-demand services).

### Statistical Analysis

Health care contacts were summarized by month and type of contact. Data were analyzed graphically to compare the development of index and follow-up contacts using monthly averages for the 3 study periods (January-June 2018, January-June 2019, and January-June 2020). Regression-based unpaired *t* tests using data on the index visit level were applied to test if the average number of follow-up contacts per index visit in March-June in 2020 was different from the number in the corresponding periods in 2018 and 2019. Pearson χ_2_^2^ tests were used to test the null hypothesis that the distribution of the types of follow-up contacts—in-person, remote (traditional), or telemedicine—was similar in these periods.

### Ethical Considerations

This research and the individual-level data compilation have been approved by the Ethical Regional Review Board in Gothenburg (Dnr: 068-18) and Swedish Ethical Review Authority (2020-02405).

## Results

### Yearly Comparisons

Table 3 displays the number of index and follow-up contacts (in total and per 1000 inhabitants) and Table 4 presents follow-up contacts per index contact (decomposed by type of contact) for each year. The total incidence of RTI consultations (index + follow-up) was the largest in 2018 (79.82 + 38.95 = almost 120 index and follow-up contacts/1000 inhabitants). The totals in 2019 and 2020 were similar (approximately 105 contacts/1000 in both years). Comparing the pandemic spring 2020 with the springs of 2018 and 2019, there were fewer index contacts in 2020 (63 vs 70-80 per 1000 inhabitants) and a larger number of follow-up visits, both per 1000 inhabitants (42 vs 36-39) and per index contact (0.66 vs 0.49-0.51).

Table 3 also shows that the share of remote contacts—with either traditional or private telemedicine providers—was larger in 2020 compared with previous years (0.26 compared with 0.08 and 0.10). Notably, the share of remote contacts with private telemedicine providers increased from 0.04 to 0.06 (which is a substantial increase in relative terms) already between 2018 and 2019. Table 3 further shows that the number of hospitalizations (per 1000 inhabitants) with relevant diagnoses was larger in 2020. However, after subtracting the hospitalizations directly related to COVID-19, the number was instead lower than that during previous years.

Table 4 shows a decomposition of follow-up contacts, both by type of index contact and by type of follow-up contact. The number of follow-up contacts per index contact was larger for remote index contacts than for in-person index contacts in all years (eg, in 2020 there were 0.60 follow-up contacts per in-person index contact, compared with 0.90 follow-up contacts per remote index contact with a traditional provider, and 0.69 follow-up contacts per on-demand index contact). In particular, the number of follow-up contacts was substantially higher for remote index contacts with traditional care providers than for the 2 other types of index contacts, which had more similar rates.

**Table 3:** Number of index and follow-up contacts by year.

Contacts per 1000 inhabitants			Year				
			2018		2019		2020
Index contacts (total)			107,330		95,955		87,125
**Index contacts/1000^a^**			79.82		70.44		63.23
	Share in-person	0.92		0.90		0.74	
	Share remote (traditional)	0.04		0.04		0.16	
	Share telemedicine	0.04		0.06		0.10	
Follow-up contacts (total)			52,370		49,399		57,253
**Follow-up contacts/1000^a^**			38.95		36.27		41.55
	Share in-person	0.63		0.62		0.46	
	Share remote (traditional)	0.34		0.34		0.47	
	Share telemedicine	0.03		0.04		0.07	
Hospitalizations/1000^a^			4.75		4.16		5.12^b^
Population^c^			1,344,685		1,362,163		1,377,826

^a^Number of contacts per 1000 inhabitants in the population.

^b^When subtracting diagnoses directly related to COVID-19, the number of hospitalizations/1000 is equal to 3.25.

^c^The population includes all individuals with a registered address in Skåne (Region Skåne) on December 31 the preceding year (according to the Swedish population register held by Statistics Sweden).

**Table 4:** Number of follow-up contacts per index contact (by type of contact).

Index and follow-up types			Year				
		2018		2019		2020	
**Index, All**							
	All	0.49		0.51		0.66	
**Index, in-person**							
	All	0.48		0.50		0.60	
	In-person	0.30		0.31		0.30	
	Remote (traditional)	0.16		0.18		0.27	
	Telemedicine	0.01		0.01		0.02	
**Index, remote (traditional)**							
	All	0.73		0.75		0.90	
	In-person	0.37		0.37		0.29	
	Remote (traditional)	0.35		0.38		0.58	
	Telemedicine	0.01		0.01		0.02	
**Index, telemedicine**							
	All	0.49		0.54		0.69	
	In-person	0.30		0.33		0.28	
	Remote (traditional)	0.05		0.06		0.14	
	Telemedicine	0.13		0.15		0.26	

### Comparison of Monthly Development of Index and Follow-Up Contacts

**Figure 1:**
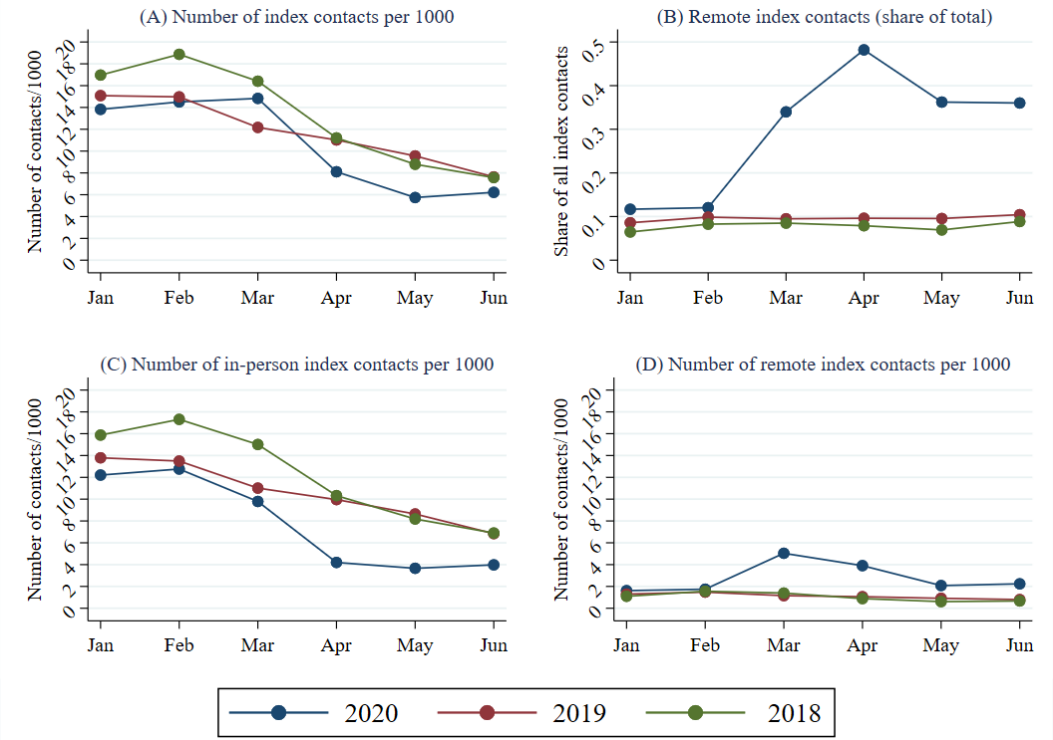
Index contacts by year, month, and type of contact. (A) The number of index contacts per 1000 inhabitants and (B) the share of the index contacts that were conducted remotely. The number of index contacts per 1000 inhabitants decomposed by (C) in-person and (D) remote contacts (independent of provider).

Figure 1 shows the development of the index contacts by month and year. Figure 1A displays all index contacts (per 1000 inhabitants) and Figure 1B shows the share of consultations that were remote contacts. The number of in-person (Figure 1C) and remote (Figure 1D) index contacts per 1000 inhabitants is also presented in the figure. Figure 1 shows that before the pandemic started, the number of index contacts in 2020 was at a similar level as in the corresponding months during previous years. From the 2018/19 graphs, we see that RTI-related index contacts usually peak in February and decrease in the following months. In 2020, these contacts instead peaked in March, and then decreased to lower levels than usual in the following months. The reduction in the number of contacts was primarily due to a reduction of in-person index contacts (Figure 1D). They increased and peaked in March before decreasing at the end of the study period. The Figure 1B shows that the share of index contacts that were conducted remotely was small and close to constant during 2018 and 2019. During 2020, the share increased substantially in March-April and then decreased, although it remained at a substantially higher level than in previous years.

**Figure 2:**
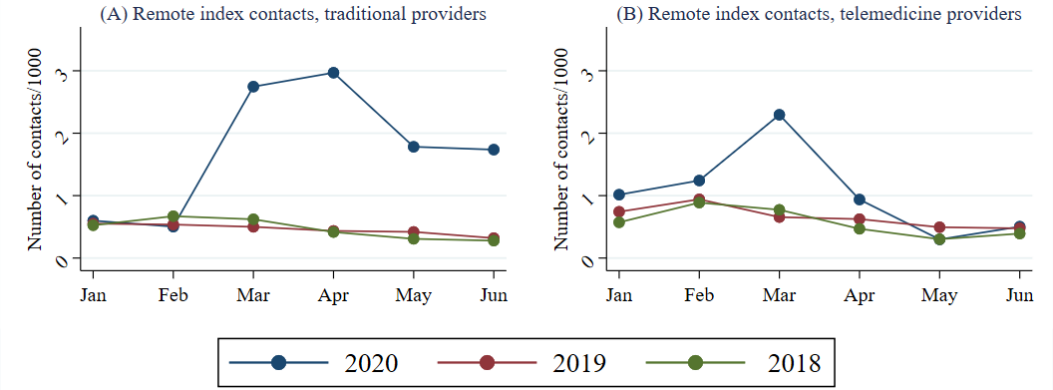
Remote index contacts by year, month, and type of provider. Graphs present the number of remote index contacts with traditional providers and remote contacts with private telemedicine providers separately.

Figure 2 displays the development of remote index contacts decomposed by type of provider (traditional provider or private telemedicine provider).

While the number of remote contacts with private telemedicine providers was slightly higher in comparison to previous years already in January 2020, the number of remote contacts with traditional providers in January to February was at the same level from 2018 to 2020. Between February and March 2020, there was a substantial increase in the number of remote contacts in comparison to previous years for both provider types. However, for private telemedicine providers, the increase was only temporary, and it was smaller (in both absolute and relative terms) than that for traditional providers. Although the number of remote index contacts with traditional primary care providers decreased slightly after April, it remained more than twice as high as in January.

**Figure 3:**
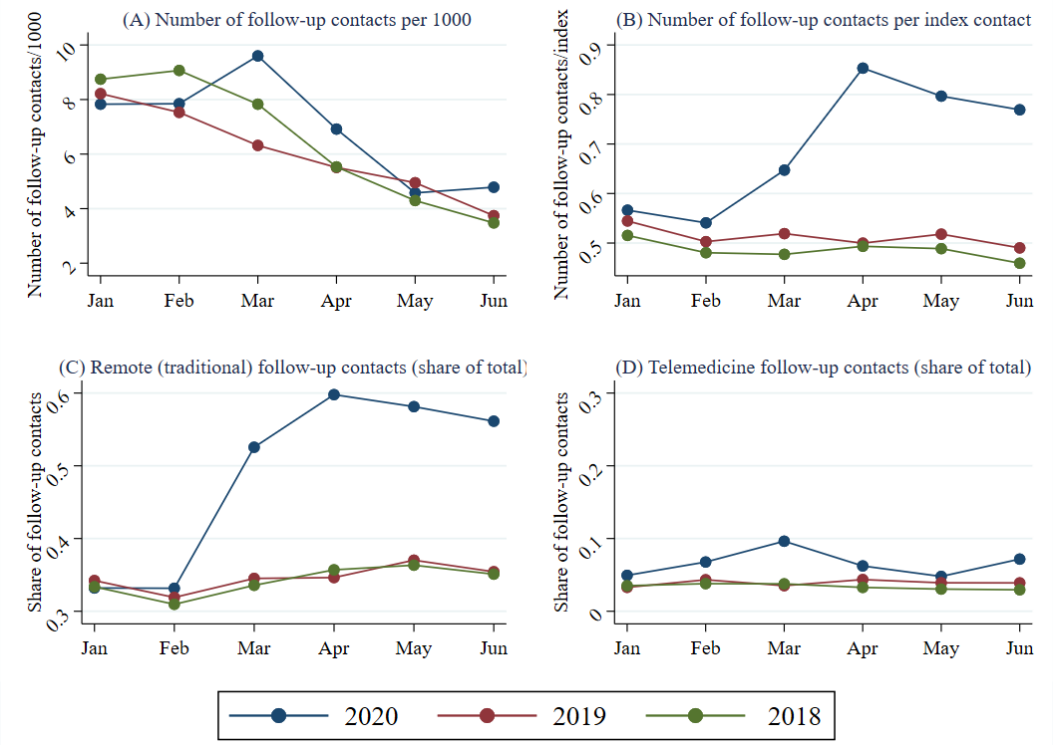
Monthly follow-up contacts, 2018-2020.

Figure 3 presents the trends of follow-up contacts per month and year. The number of follow-up contacts per 1000 inhabitants in January-February was similar in 2020 and in 2019, but higher in 2018 (because the number of index contacts was higher; Figure 3A). During March and April 2020, the number of follow-up contacts was higher in 2020 than in the corresponding months of 2018 and 2019. The development in the number of follow-up contacts per index contact during 2020 deviated substantially from previous years from March onward (Figure 3B). The share of follow-up contacts conducted remotely increased, in particular for the traditional primary care providers (Figure 3C), but to some degree also for private telemedicine providers (Figure 3D).

### Differences in Type of Follow-Up Contacts Per Index Contact

Table 5 presents the difference in the average number of follow-up contacts per index contact between the pandemic period (March to June 2020) and the corresponding period in 2018 and 2019. Every row represents a type of index consultation, and each column represents a type of follow-up contact. The category *All* includes in-person contacts and remote contacts with either a telemedicine or traditional primary care provider. These results provide an overview of how COVID-19 changed the number and composition of follow-up contacts per index contact. The fifth column presents the Pearson χ_2_^2^ statistics testing whether the composition of the follow-ups in 2020 is statistically different from the previous years.

**Table 5:** Difference in the number of contacts within 30 days per index contact.

Type of index contact	Type of follow-up^a^						
	All	In-person	Remote (traditional)	Telemedicine	χ_2_^2^		
All	0.25 (<0.001)	–0.032 (<0.001)	0.24 (<0.001)	0.037 (<0.001)	5437 (<0.001)		
In-person	0.19 (<0.001)	–0.029 (<0.001)	0.21 (<0.001)	0.012 (<0.001)	2775 (<0.001)		
Remote (traditional)	0.17 (<0.001)	–0.080 (<0.001)	0.24 (<0.001)	0.014 (<0.001)	372 (<0.001)		
Telemedicine	0.20 (<0.001)	–0.0087 (<0.001)	0.13 (<0.001)	0.16 (<0.001)	645 (<0.001)		

^a^*P* values are in parenthesis (see text for detailed explanation).

The differences in the average number of follow-up contacts per index are obtained from linear regression models. Each coefficient represents the difference in the average number of follow-up contacts per index contact between March to June in 2020 and the same period in 2018 and 2019. *P* values from hypothesis tests of the coefficient being equal to 0 are presented in parenthesis. These *P* values, which are based on robust standard errors, indicate that all differences are significantly different from 0. The last column presents results from Pearson χ_2_^2^ tests of the null hypothesis that the distribution of the types of follow-up contacts—in-person, remote (traditional), or telemedicine—from March to June 2020 was the same as in the corresponding periods in 2018 and 2019. These *P* values indicate that for all types of index contacts, the distribution of follow-up contacts in 2020 was different from that in previous years.

The first row, which considers all contacts irrespective of type, shows that the average number of follow-up contacts per index contact increased by 0.25 in 2020 compared with the same period in previous years. The composition of follow-up contacts also changed, with fewer in-person and more remote contacts. The increase was especially strong for follow-up contacts with traditional providers: the increase of this type of contact was of similar size as the total increase. The increase of 0.037 follow-up contacts with private telemedicine providers thus almost offset the decrease of 0.032 in-person follow-up contacts.

Looking at the results by type of index contact, the total number of follow-up contacts per index contact (subcolumn All) increased by a similar amount for all types of index contacts. (Note that the change for the All category [0.25] is larger than the changes for each type of index contact [0.17-0.20]). This is due to the change in the composition of index contacts illustrated in Tables 3 and 4 and Figure 1. The shift from in-person to remote follow-up contacts was also visible for all index contact types. The overall increase for index contacts with a traditional provider was primarily due to an increase in remote follow-up contacts with the same kind of provider; follow-up contacts with private telemedicine providers only increased marginally.

The increase in the total number of follow-up contacts per private telemedicine index (presented in the fourth row) is of similar size as the increase in telemedicine follow-up contacts. Thus, the increase in remote follow-ups with traditional providers was offset by a decrease in in-person follow-up contacts.

## Discussion

### Principal Findings

The total number of index contacts for RTIs decreased in spring 2020 compared with the corresponding periods in 2018 and 2019. This pattern may partly be explained by the governmental decision in the spring of 2020 to temporarily remove the mandatory sick certificates necessary after the first sickness week [17], certificates that usually needed an in-person contact with a physician prior to the pandemic. Another possible explanation relates to the initial shortage of SARS-CoV-2 test capacity. Patients with light/moderate RTI symptoms might have followed the advice to stay in quarantine and not contact the health care service unless they developed severe symptoms. Social distancing and hygiene measures probably also lead to a general decrease in viral infections such as influenza [18]. Given that the total incidence of RTI contacts was similar in the springs of 2019 and 2020, one possible interpretation of the decrease in index visits in 2020 could be that an increasing load of follow-up contacts during the pandemic spring displaced new patients (new index contacts). However, the monthly trends shown in Figures 1 and 3 point against this interpretation, as both index and follow-up contacts peaked during the same month (March) and index contacts decreased more than the increase in follow-up contacts in April. In other words, the peak in follow-up contacts took place well before the drop in index contacts.

The share of index contacts conducted remotely increased substantially, particularly for traditional care providers. The increase in remote index contacts peaked in March 2020 for both traditional and private telemedicine providers and remained high for traditional care providers until the end of our study period (June 2020). With regard to follow-up contacts, the pandemic spring 2020 was associated with both an increase in the number of follow-up contacts per index and an increasing share of remote follow-up contacts, regardless of whether the index contact was conducted in-person or remotely.

Already before the pandemic, traditional care providers provided more follow-up contacts after remote index contacts than after in-person index contacts compared with private telemedicine providers. As the composition of the index contacts with traditional providers during the pandemic shifted toward a larger share of remote contacts, part of the increase in the total number of follow-up contacts is therefore probably due to traditional providers’ habit of offering more follow-up contacts after remote index contacts. Furthermore, most patients with chronic diseases (eg, heart failure or chronic obstructive pulmonary disease) were assessed remotely during the spring of 2020 but needed increased attention in case of simultaneous symptoms for RTIs. Providers may also have been more generous with offering follow-up contacts due to the uncertainty surrounding diagnoses during the pandemic spring, before testing for SARS-CoV-2 was widely available. During the same period, elderly were generally more cautious in booking in-person contacts and preferred to contact their physician remotely if possible.

The difference in the number of follow-up contacts per index contact between 2020 and the previous years shown in Table 5 cannot be interpreted as only being due to the changes in utilization patterns induced by COVID-19. There may have also been an ongoing secular time trend in utilization. For example, there was a larger number of follow-up contacts per index contact in January-February 2020 (ie, before the pandemic) than in the corresponding months of previous years. However, an analysis that accounts for the influence of such secular trends yields similar results (Multimedia Appendix 2). The exception is that a substantial share of the increase in the number of follow-up contacts per private telemedicine index contact is likely due to such trends.

Whether the increase in follow-up contacts during the first wave of pandemic is a concern for future policy depends on the reasons behind this increase. If this reflects a change in the type of managed complaints or the severity of the cases due to the pandemic, the pattern is likely only relevant for a pandemic state. By contrast, if the trend primarily reflects a change in practice—among providers and patients—when it comes to adopting a digital/remote-first approach, the change may have lasting impacts also after the pandemic. While the increase in consultations with private telemedicine providers is demand driven, the increase in the number of remote contacts within traditional providers likely reflects a change in practice among providers (rather than among patients). The larger number of follow-up contacts for remote index contacts could possibly indicate that management of RTIs might be challenging using a remote contact for the initial symptom assessment, leading to more follow-up contacts to ensure patient safety.

### Limitations

The rich and detailed administrative data are one of the main strengths of the study. Using different register sources, we managed to collect information from all telemedicine providers available during the period studied. The study design, including data for all inhabitants in Skåne representing all sociodemographic and geographic varieties, indicates a high generalizability of the results.

There are also limitations of these data. We have no available data on symptom severity that can confirm or dismiss the hypothesis that the severity of symptoms managed by different providers may differ. There is variation in the number of registered diagnoses between data sources, providers, and types of contacts (see Multimedia Appendix 2 for more detailed information). For example, there is no information on registered diagnoses for 2 minor private telemedicine providers before June 2019. The main issue is, however, that traditional providers are less prone to register diagnoses during a remote contact.

Missing information on diagnoses implies that there is a risk that a contact is erroneously classified as an index contact because an actual RTI episode during the wash-out period has not been registered as such. It may also lead to an underreporting of actual index contacts if there is no registered diagnosis at all during an episode. Diagnosis registration is an administrative task that physicians may fail to perform consistently for follow-up contacts, mainly due to lack of time but also because the diagnosis is already documented in the electronic medical journal after the initial contact. We estimate these possible registration bias as a minor risk, due to administrative demands in the study region. Specifically, care givers in the region have financial incentives to register diagnoses, as the case-mix adjustment formula used in the regional reimbursement model relies on diagnoses registered in the electronic medical journal. Simply put, every unique diagnosis registered for a patient increases the expected reimbursement.

Because of the risk that physicians may fail to consistently register diagnoses for follow-up contacts, we included all follow-up contacts regardless of diagnosis. This means that some of the follow-up contacts may not have had any association with the index contact. However, as the same approach was used for all years and types of contact, the effects on the comparisons are likely small.

### Comparison With Prior Work

Prior to the SARS-CoV-2 pandemic, telemedicine was implemented at a low pace by traditional providers, both in Sweden and internationally [4,19]. During the pandemic outbreak, a framework for telemedicine was defined and updated [14] and stakeholders were encouraged to implement and integrate it within the national health care systems [20]. It has also been argued that the fast implementation of telemedicine might be one of the most positive effects of the pandemic [21], given that it succeeds in substituting for routine care or complementing in-person care [22]. Telemedicine undoubtedly plays an important role in outbreak containment, as most patients with mild RTIs can be managed remotely [14]. However, despite obvious advantages with remote care for RTIs (such as time saving, lack of travel costs, or lower risk for viral spread), it is also possible that larger accessibility to remote care may lead to patients pushing for unnecessary follow-up contacts or for assessment of uncomplicated medical complaints that usually do not require a physician contact. The private telemedicine providers may be more responsive to such demands from patients, as they are reimbursed per visit. However, most of the increased remote contacts in this study were delivered by traditional care providers, whose reimbursement does not depend on the number of visits. This suggests that the patterns are unlikely to reflect increases of unnecessary care. However, it might still be the case that increased accessibility (by phone or chats) leads to increased pressure on the nurses handling requests from patients. A recent study in a British setting showed that the “telephone-first approach” resulted in more phone calls, fewer physical consultations, and on average, more time spent consulting [23] and found no evidence that this type of care is cost saving.

The number of hospitalizations for RTI-relevant diagnoses was higher in 2020 compared with previous years, but when excluding diagnoses directly related to COVID-19, the number of hospitalizations was substantially lower in 2020. Previous studies have shown that the number of hospitalizations for acute cardiovascular conditions [24] or stroke [25] decreased during the pandemic, possibly due to an altered pattern of emergency care seeking in the population or lockdown rules. These mechanisms are possibly relevant also for the patterns in our study region, although it may also relate to decreased spread of RTIs following social distancing policies.

### Conclusions

Compared with 2018 and 2019, there were fewer index consultations for RTIs but more follow-up contacts, both per 1000 inhabitants and per index contact, in 2020. The share of both index and follow-up contacts that were conducted remotely increased, in particular for contacts with traditional care providers. The share of contacts supplied by private telemedicine providers only increased temporarily. Hence, it seems that the COVID-19 pandemic contributed to an increased number of remote physician contacts and follow-up contacts for RTIs. This could indicate that patients with RTI needed to be reassessed more often when the physician did not have the possibility to examine the patient in-person. Further studies are needed to study the reasons behind the increase in remote contacts, and if it will last after the pandemic, and more clinical guidelines for remote assessments of RTI are warranted.

## Appendix

Multimedia Appendix 1 Data Source and Variable Definitions.

Multimedia Appendix 2 Difference-in-Differences Analysis.

## Abbreviations

RSVD: Region Skånes Vårddatabas
RTI: respiratory tract infection
