# Supplementary material for: Trends in Remote Health Care Consumption in Sweden: Comparison Before and During the First Wave of the COVID-19 Pandemic
Source: JMIR Hum Factors. 2022 Feb 2;9(1):e33034. doi: 10.2196/33034 (PMC8812677; doi:10.2196/33034)
Supplement: Multimedia Appendix 1 [file humanfactors_v9i1e33034_app1.docx]

Appendix 1: Data source and variable definitions

Table 1: Data sources per private telemedicine provider.

| Provider | Source 1 | Source 2 | Source 3 |
| --- | --- | --- | --- |
| Kry | Jönköping | Sörmland | RSVD^a^ |
|  | (170701-200430) | (190228-) | (181204-) |
| Capio Go | RSVD^a^ | Jönköping | Sörmland |
|  | (170701-180531) | (180531-200430) | (200501-) |
| Min Doktor | Jönköping | Sörmland |  |
|  | (170701-190729) | (190801-) |  |
| Doktor.se | RS-UL^b^ | Sörmland |  |
|  | (170701-190603) | (190604-) |  |
| Doktor 24 | Jönköping | Sörmland |  |
|  | (180525-190516) | (190517-) |  |
| Medicoo | Jönköping |  |  |
|  | (170701-190412) |  |  |
| Accumbo | Sörmland |  |  |
|  | (191001-) |  |  |

^a^RSVD = Region Skånes Vård Databas (intra-regional health care register)

^b^RS-UL = Extra-regional health care register

*Note: RSDV includes up to 8 diagnoses. RS-UL does not include diagnosis information. The number of registered diagnoses is 1 in Sörmland and up to 5 in Jönköping.*

Figure 1: Flow chart of data sources for physician consultations.

N= total number physician consultations.

*Percentage of consultations with no diagnosis registered.

Data on contacts are from the regional health authority’s care register “Region Skånes Vårddatabas” (RSVD), a register of extra-regional care contacts of inhabitants in Skåne (RS-UL), and from regional health authorities in Region Sörmland and Region Jönköping. The flow chart in Figure 1 illustrates how data on physician consultations are composed from various sources. Table 1 presents the data source for each private telemedicine provider and time period. The extent to which these sources include registered diagnoses vary. The table and figure are jointly informative about missing information for each type of contact and data source. The numbers in each box correspond to the number of registered contacts during our complete study period (July 2018 to July 2021). The share of consultations with no registered diagnosis is presented in parenthesis. The share varies substantially between data sources.

Definitions of provider type, contact type and data sources are spelled out below:

**Traditional providers** include all primary care centers and hospital emergency rooms. A primary care center is a unit operating within the patient choice system in Region Skåne. The definition includes units operating during both office hours and out of office hours. The definition also includes primary care centers that are operated by companies also providing direct-to-consumer telemedicine (DCT) services outside the patient choice system, .i.e., Capio and KRY. Primary care centers are primarily reimbursed based on a risk-adjusted capitation.

An ***in-person contact*** is defined as:

- a physician contact at a primary care center and type of contact registered as ‘new contact’ or ‘follow-up contact’ (coded as NY, ÅB in RSVD)
- a physician contact at a hospital emergency room, registered as ‘new contact’, ‘follow-up contact’, or as a ‘contact with an (internal) consultant’ (coded as NY, ÅB, KI, KO in RSVD) , or any registered in-patient episode at a hospital emergency room.

A ***remote contact*** with a traditional provider is defined as a qualified telephone contact or video contact (coded as KT, VK in RSVD) with a primary care center, or a hospital emergency room. Such contacts include qualified telephone contacts, video contacts and asynchronous chat contacts. Remote contacts (coded as KT, VK in RSVD with primary care units operated by private telemedicine providers are included among these contacts. Contacts with the digital platform (i.e., DCT contacts) are not included here.

In-person and remote contacts with traditional providers are all obtained from RSVD. Contacts registered in RSVD have up 8 registered diagnoses.

**Private telemedicine providers** include all providers offering DCT (or on-demand) telemedicine services outside the patient choice system. These providers are Kry, Capio Go, Min Doktor, Doktor.se, Doktor 24, Medicoo, and Accumbo. (See Table 1 for relevant providers and data sources). These operate on a fee-for-service basis.

Due to the regulation in place these providers may be located in any region in Sweden. Consequently, the information on these contacts is obtained from various sources.

Information on contacts with private telemedicine providers located in Skåne is obtained from RSVD and a register of extra-regional care contacts of inhabitants in Skåne (RS-UL). Information on other contacts is obtained from regional health registers held by Region Sörmland and Region Jönköping (We discuss an exception below). Table 1 presents the source per provider and time period. Below we provide further information.

- Capio Go and Kry have for parts of the study period been operated from Region Skåne. For these periods at least some of DCT contacts have been registered in RSVD. The register differentiates between a DCT contact with Kry or Capio Go and an in-person or remote contact with the primary care center operated by Kry or Capio. This post only includes DCT contacts.
  - Contacts with Capio Go registered in Skåne have no registered diagnoses.
  - 57.96% of all contacts with Kry registered in Skåne have no registered diagnosis.
- Both Kry and Capio Go have also been located in other regions during the study period.
- Doktor.se was located in Sörmland already at the start of the study period. Contacts at Doktor.se were not registered in the local register at Sörmland used in this study until 2019-07-29. RS-UL includes all contacts with providers in other regions. These contacts include limited information (date and type of care giver). Registered physician contacts in RS-UL that are not matched (by day and individual) with register data from Sörmland are assumed to be contacts with Doctor.se (up to 20190729). These contacts have no registered diagnoses.
- Contacts with telemedicine providers in Sörmland have up 1 diagnosis registered, 0.16% has no registered diagnosis. (See Table 1 for relevant providers and time periods).
- Contacts with telemedicine providers in Jönköping have up 5 diagnoses registered, 0.45% has no registered diagnosis. (See Table 1 for relevant providers and time periods).
