# Supplementary material for: Trends in Remote Health Care Consumption in Sweden: Comparison Before and During the First Wave of the COVID-19 Pandemic
Source: JMIR Hum Factors. 2022 Feb 2;9(1):e33034. doi: 10.2196/33034 (PMC8812677; doi:10.2196/33034)
Supplement: Multimedia Appendix 2 [file humanfactors_v9i1e33034_app2.docx]

**Appendix 2: Difference-in-Differences Analysis**

Table 1 decomposes follow-up contacts by type, both in terms of the type of index contact and type of follow-up contact. The table presents regression coefficients representing the differences in contacts per index contact between i) Jan-Feb 2020 and Jan-Feb 2018/2019 (D0), ii) March-June 2020 and March-June 2018/2019 (D1), and iii) the difference between D1 and D0 (i.e. a difference-in-difference model). That is, the first two rows in each panel present $\alpha_{2}$ from the following regression using index contacts that took place in January and February in the first row and index contacts that took place in the period from March to June in the second row:

$y_{i}=\alpha_{1}+\alpha_{2}I\left( 2020 \right)_{i}+\epsilon_{i}$ Eq(1)

where $I\left( 2020 \right)_{i}$ is a dummy indicator equal to one if the index contact took place in 2020 (in contrast to 2018 or 2019), $\alpha_{1}$ is equal to a constant and $\epsilon_{i}$ is an error term. The second row corresponds to the results presented in Table 4 in the results section in the main manuscript. This comparison, the difference between 2020 and the two previous years for the period between March to June, cannot be interpreted as only being due to the changes in utilization patterns induced by covid-19. There was also an ongoing secular time trend in utilization. For example, there was a larger number of follow-up contacts per index contact in Jan-Feb 2020, i.e., before the pandemic, than in the corresponding months earlier years (as indicated by the results in the first row in the fourth panel).

The difference-in-difference analysis presented in the third row in each panel account for the influence of the secular trends by reducing the difference in March to June by the difference in Jan and February. Assuming that the difference before the pandemic capture the secular trend, the difference-in-difference analysis provides a better estimate of how covid-19 changed the number of follow-ups per index contact. The third row in each panel presents the difference-in-difference coefficients, $\beta_{4}$ from the following regression:

$y_{i}=\beta_{1}+\beta_{2}*I\left( 2020 \right)_{i}+\beta_{3}*I\left( MarchJune \right)_{i}+\beta_{4}*I\left( 2020 \right)_{i}*I\left( MarchJune \right)_{i}+e_{i}$ Eq(2)

where $I\left( MarchJune \right)$ is an indicator equal to one if the index contact took place in the period between March to June, $\beta_{1}$-$\beta_{4}$ are regression coefficients and $e$ an error term.

Every panel in the Table 1 represents one type of index contact, and every column represent one type of follow-up contact. The category ALL includes in-person contacts and remote contacts with either a private telemedicine provider or a traditional primary care provider. The coefficients for D0 shows that the differences between the Jan-Feb periods in 2020 and 2018/19 were small, except for telemedicine contacts. The significant D0 coefficient for telemedicine implies that the number of follow-up contacts per index contacts was 0.12 larger in 2020 for Jan & Feb. The D1 coefficients for all type of follow-up contacts are all large and significant confirming that there was a substantial and significant difference between April-June in 2020 and the earlier two years. Thus, this analysis indicates that the increase in the number of follow-up contacts per private telemedicine index consultation reported in table 4 is overestimated. A share of this increase is due to the secular trend. For the other outcomes, the results in table 4 are similar to the results of the analysis accounting for the secular trend. Notably, the main coefficient for the difference between the differences D1 and D0 confirm that there was an increase in the number of follow-up contacts per index contact as a result of the pandemic. The total increase was primarily an increase in remote follow-up contacts with traditional primary care providers.

Table 1: Difference in the number of follow-up contacts per index contact

| Difference | Follow-up (Index: All Contacts) | | | | |
| --- | --- | --- | --- | --- | --- |
|  | | All | In-person | Remote, (trad) | Telemedicine |
| All D0 | | 0.044 | 0.013 | 0.018 | 0.013 |
|  | | (<0.001) | 0.001) | (<0.001) | (<0.001) |
| All D1 | | 0.25 | -0.032 | 0.24 | 0.037 |
|  | | (<0.001) | (<0.001) | (<0.001) | (<0.001) |
| All D1-D0 | | 0.20 | -0.045 | 0.23 | 0.024 |
|  | | (<0.001) | (<0.001) | (<0.001) | (<0.001) |
|  | | Follow-up (Index: in-person) | | | |
|  | | All | In-person | Remote, (trad) | Telemedicine |
| in-person D0 | | 0.036 | 0.012 | 0.019 | 0.0051 |
|  | | (<0.001) | (0.005) | (<0.001) | (<0.001) |
| in-person D1 | | 0.19 | -0.029 | 0.21 | 0.012 |
|  | | (<0.001) | (<0.001) | (<0.001) | (<0.001) |
| in-person D1-D0 | | 0.16 | -0.040 | 0.19 | 0.0072 |
|  | | (<0.001) | (<0.001) | (<0.001) | (<0.001) |
|  | | Follow-up (Index: remote, traditional) | | | |
|  | | All | In-person | Remote, (trad) | Telemedicine |
| remote (trad) D0 | | 0.031 | -0.021 | 0.048 | 0.0044 |
|  | | (0.398) | (0.306) | (0.058) | (0.183) |
| remote (trad) D1 | | 0.17 | -0.08 | 0.24 | 0.014 |
|  | | (<0.001) | (<0.001) | (<0.001) | (<0.001) |
| remote (trad) D1-D0 | | 0.14 | -0.058 | 0.19 | 0.0095 |
|  | | (0.001) | (0.013) | (<0.001) | (0.019) |
|  | | Follow-up (Index: telemedicine) | | | |
|  | | All | In-person | Remote, (trad) | Telemedicine |
| telemedicine D0 | | 0.12 | 0.043 | 0.02 | 0.055 |
|  | | (<0.001) | (0.005) | (0.009) | (<0.001) |
| telemedicine D1 | | 0.20 | -0.087 | 0.13 | 0.16 |
|  | | (<0.001) | (<0.001) | (<0.001) | (<0.001) |
| telemedicine D1-D0 | | 0.083 | -0.13 | 0.11 | 0.10 |
|  | | (0.005) | (<0.001) | (<0.001) | (<0.001) |

*Note: The first two rows in each panel show regression coefficients indicating i) the difference in the number of follow-up contacts per index contact in January and February between 2020 and 2019/2018 (D0), ii) the difference in the number of follow-up contacts per index contact in March to June between 2020 and 2019/2018 (D1). The third row in each panel shows difference-in-difference coefficients comparing the difference in March to June with the difference in January and February (D1-D0). P-values based on robust standard errors are presented in parenthesis.*
